# Recombinant CsHscB of carcinogenic liver fluke *Clonorchis sinensis* induces IL-10 production by binding with TLR2

**DOI:** 10.1371/journal.pntd.0008643

**Published:** 2020-10-12

**Authors:** Chao Yan, Fan Fang, Yu-Zhao Zhang, Xin Dong, Jing Wu, Hai-Liang Liu, Chun-Yang Fan, Stephane Koda, Bei-Bei Zhang, Qian Yu, Liang Wang, Yu-Gang Wang, Jia-Xu Chen, Kui-Yang Zheng

**Affiliations:** 1 Jiangsu Key Laboratory of Immunity and Metabolism, Department of Pathogenic Biology and Immunology, Laboratory of Infection and Immunity, Xuzhou Medical University, Xuzhou, P. R. China; 2 National Experimental Demonstration Center for Basic Medicine Education, Department of Clinical Medicine, Xuzhou Medical University, Xuzhou, P. R. China; 3 CapitalBio Technology Inc., Beijing, P.R. China; 4 College of Bioinformatics, Xuzhou Medical University, Xuzhou, P. R. China; 5 National Institute of Parasitic Diseases, Chinese Center for Disease Control and Prevention, Key Laboratory of Parasite and Vector Biology, Ministry of Health, WHO Collaborating Center of Malaria, Schistosomiasis, and Filariasis, Shanghai, P. R. China; Istituto Superiore di Sanità, ITALY

## Abstract

**Background:**

*Clonorchis sinensis*, a fluke dwelling in the intrahepatic bile ducts causes clonorchiasis, which affect about 15 million people wide-distributed in eastern Asia. During *C*. *sinensis* infection, worm-host interaction results in activation of patterns recognition receptors (PRRs) such as Toll-like receptors (TLRs) and further triggers immune responses, which determines the outcome of the infection. However, the mechanisms by which pathogen-associated molecules patterns from *C*. *sinensis* interact with TLRs were poorly understood. In the present study, we assumed that the molecules from *C*. *sinensis* may regulate host immune responses via TLR2 signaling pathway.

**Methodology/Principal findings:**

In the present study, we have identified a ~34 kDa CsHscB from *C*. *sinensis* which physically bound with TLR2 as demonstrated by molecular docking and pull-down assay. We also found that recombinant CsHscB (rCsHscB) potently activates macrophage to express various proteins including TLR2, CD80, MHCII, and cytokines like IL-6, TNF-α, and IL-10, but rCsHscB failed to induce IL-10 in macrophages from *Tlr2*^-/-^ mice. Moreover, ERK1/2 activation was required for rCsHscB-induced IL-10 production in macrophages. *In vivo* study revealed that rCsHscB triggered a high production of IL-10 in the wild-type (WT) but not in *Tlr2*^-/-^ mice. Consistently, the phosphorylation of ERK1/2 was also attenuated in *Tlr2*^-/-^ mice compared to the WT mice, after the treatment with rCsHscB.

**Conclusions/Significance:**

Our data thus demonstrate that rCsHscB from *C*. *sinensis* interacts with TLR2 to be endowed with immune regulatory activities, and may have some therapeutic implications in future beyond parasitology.

## Author summary

*Clonorchis sinensis* is a kind of liver fluke whose infection can cause inflammation and, fibrosis around the bile ducts. As type I biological agents (carcinogens) Type, it can also cause bile ductal cancer. However, the mechanisms by which the parasite interacts with host cells and further induce serious sequences are poorly understood. Here, we found a~34 kDa rCsHscB from *C*. *sinensis* that can bind with TLR2, a well-known pattern recognition receptor. Importantly, we found that rCsHscB could lead to the activation of macrophage and induce a strong production of cytokines such as IL-10 and IL-6 through the TLR2-mediated signaling pathway. Besides, the animal study demonstrated that rCsHscB induce a high production of IL-10 in the wild-type (WT) but not in *Tlr2*^-/-^ mice. These data showed that rCsHscB from *C*. *sinensis* interacted with TLR2 have immune-regulatory abilities, which can be used for other inflammatory disorders in the future beyond parasitology.

## Introduction

During helminth infection, the interaction of the parasite with his host triggers host immune responses which ultimately drives the resistance to infection or immune evades accompanying the course of immunopathogenesis. From this perspective, type 2 immune responses including IL-4, IL-5, IL-9, and IL-13 secreted by ILC2, Th2, or alternatively activated macrophage (AAM or M2) are typically considered as protective immunity against helminths to results in parasite expulsion ultimately [1]. However, the regulatory cells (Treg, Breg, ILCreg, M2c, etc) can produce the regulatory cytokines (IL-10, etc) to ameliorate the bias of type II immune responses, which appears to be mainly responsible for the worms’ survival with the limited immunological damages and further establishment of chronic infection [2]. Previous studies have demonstrated that MAPK (such as ERK, p38) and NF-κB signaling (NF-κB p50 homodimers) contribute to the mechanisms that control the production of IL-10 [3, 4]. Nevertheless, the mechanisms by which the complex immune responses are initiated and finely-orchestrated remain poorly elucidated.

Toll-like receptors represent one of the most important patterns recognition receptors that sense the conserved pathogen products (also called pathogen-associated molecular patterns, PAMPs) from worms or alarming (also called danger-associated molecular patterns, DAMPs) sourced from damage tissues in the early event of infection. For example, TLR2 interacts with TLR1 or TLR6 to recognize triacylated or diacylated lipoproteins, respectively, and thereby activate signal transduction cascades to result in the expression of pro-inflammatory or anti-inflammatory mediator genes [5–7]. So far several TLR2 ligands from *Schistosome mamansoni*, *Wolbachia*-endosymbiotic bacteria of *Brugia malayi* have been identified and demonstrated as potent immune regulators to determine the polarization of immune and even the outcome of helminth infection. For example, lysophosphatidylserine (Lyso-PS) from *S*. *mansoni* bound with TLR2 on dendritic cells allows DC to train IL-10 producing Tregs, which enables the long term survival of the parasite, as well as ameliorates of immune-pathogenesis due to polarized type 2 immune responses [8]. Diacyl WoLP sourced from *Wolbachia* induces dendritic cell maturation and activation as well as drives CD4+ T cell polarization and antibody switching in a TLR2-dependent manner [9].

Clonorchiasis caused by *Clonorchis sinensis* remains a major parasitic disease in eastern Asia such as China, Korea, Vietnam, and eastern Rusia [10]. There are approximately 15 million people infected worldwide whereas 12.5 million people are distributed in China, posing a severe public health issue in these regions [11]. The adult worms dwelling in the intrahepatic bile duct cause cholelithiasis, cholangitis, cholecystitis, biliary fibrosis, and even cirrhosis due to its long-term survival. Additionally, chronic infection with this fluke has been shown to cause cholangiocarcinoma (CCA) and *C*. *sinensis* is now defined as Group 1 human biological agents (carcinogens) by the International Agency of Research on Cancer (IARC) due to sufficient pieces of evidence in human [12, 13]. Previous studies have shown that the components of *C*. *sinensis* excretory/secretory products (ESPs) and crude antigen (CA) can potently induce a type 2 or a mix type1/type2 immune responses *in vitro* [14–16]. During the *in vivo* study of *C*. *sinensis* infection, the interaction between worms and host immune cells also potently drives type I immune responses, however, type 2 immune responses become more prevalent after worms are well-developed in susceptible hosts [17]. Furthermore, our previous study also showed that the expression of TLR2 was dramatically changed with the prolonged infection, which suggested that TLR2 might be involved in this dramatically immunological change [18]. The mechanisms that account for this phenotypic shifting are poorly understood so far. Therefore, we assumed that at least one component from *C*. *sinensis* could regulate host immune responses via TLR2 signaling pathway, which may orchestrate the outcomes of *C*. *sinensis* infection. In view of this background, we sought to identify the molecules from *C*. *sinensis* that were responsible for the activation of TLR2 and to investigate its possible effects on the activation of macrophage. In our present study, we identified a rCsHscB interacted with TLR2 acting as an unidentified TLR2 agonist that induces the activation of macrophage secreting high levels of pro-inflammatory and anti-inflammatory cytokines in a TLR2-dependent manner. Our study will contribute to a better understanding of the interaction between the *C*. *sinensis* and host cells. Besides, given regulatory immune capacities of rCsHscB, our study also provides an alternative therapeutic approach for implications beyond parasitology.

## Materials and methods

### Ethics

Animal care and all experimental performing in this study were confirmed to the guidelines of the National Laboratory Animal Center. The main procedures and protocol were reviewed and approved by the Animal Care and Use Committee of Xuzhou Medical University License (2016-SK-03).

### Mice

Male C57BL/6 mice (specific pathogen-free, SPF) aged 8 weeks (20-22g) were purchased from the Beijing Vital River Laboratory Animal Technology Co. Ltd. *Tlr2*^-/-^ mice were bought from Shanghai Model Organisms Center, Inc., China. All the mice were group-housed in a specific pathogen-free condition with the temperature-control room (25 °C). All mice were given standard chow diet and tap water *ad libitum*.

To obtain *C*. *sinensis*-positive sera, BALB/c mice were orally infected by 45 metacercariae and the mice were sacrificed on 28 days and 56 days post-infection (*p*.*i*.), the sera from *C*. *sinensis*-infected mice and no-infected mice were collected for further use.

Mice were immunized with around 10 μg of *C*. *sinensis* crude antigen in IFA. Two booster doses in IFA were injected in 15 days interval. Titers of antibodies against *C*. *sinensis* crude antigen were determined by ELISA.

### Preparation of rCsHscB and control protein

rCsHscB and control protein (only His-tagged protein encoded by pET-28a vector without CsHscB open reading frame)was routinely expressed by *E*. *coli* (Ec). In brief, The coding region of CsHscB was cloned into expression vector pET-28a (+), and then the recombinant construct of CsHscB (pET-28a/CsHscB) as well as the control vectors (without CsHscB open reading frame, pET-28a-His-tagged) were transformed into *E*. *coli* BL21 (DE3). The recombined CsHscB was induced by 1 mM Isopropyl β-D-1-Thiogalactopyranoside (IPTG) after the culture had reached 0.6 of OD 600. rCsHscB was then purified by nickel-affinity and ion-exchange chromatography.

### Development of specific rCsHscB polyclonal antibody

The antibody to the rCsHscB protein was generated in rabbits that were maintained in the animal house facility of Xuzhou Medical University. In brief, rabbits were immunized with around 10 μg of rCsHscB in IFA. Two booster doses in IFA were injected in 15 days interval. After measuring the rCsHscB-specific Ab titer by ELISA, animals were sacrificed at day 45 to collect and separate sera. The poly-antibody against rCsHscB was purified by metal affinity chromatography. In an immunoblot, the Ab raised against the rCsHscB protein specifically recognized a single band of ~36 kDa.

### Immunohistochemistry

rCsHscB was stained on paraffin-embedded adult worm *C*. *sinensis* by immunohistochemistry using the affinity-purified anti-rCsHscB antibody. Reactivity was detected using Dako Detection System ((Dako, Glostrup, Denmark). Sections were counterstained with hematoxylin and photographed by a microscope.

### Cell culture and stimulation

Mouse mononuclear macrophage leukemia cells RAW264.7with 5~10 passages were cultured in DMEM (Hyclone, US) containing 10% fetal bovine serum (FBS) (Serana, AUS), 1% penicillin/streptomycin (Beyotine, China) in a humidified atmosphere with 5% CO_2_ at 37°C. RAW264.7 cells were stimulated by rCsHscB (5~20 μg/ml) for 6 h, 12 h, and 24 h. Supernatants were collected for assessing the concentrations of cytokines using ELISA. For TLR2 blocking assay, RAW 264.7 cells were pretreated with MAb-mTLR2 (2 μg/ml) or isotype (Invivogen, US) for 2 h. The cells were then stimulated by rCsHscB (20 μg/ml) or Pam_3_CSK_4_ (200 ng/ml) (Invivogen, US) for 24 h in a humidified atmosphere with 5% CO_2_ at 37°C. The supernatants and cultured cells were collected for flow cytometry assays for ELISA, respectively. For evaluation of the toxicity of rCsHscB, the activities of LDH in the medium of cultured cells were determined by using a commercial LDH colorimetric assay kit (Abcam, ab102526).

Bone marrow cells were obtained from the long bones of 8- to 10-week-old C57BL/6 mice (WT or *Tlr2*^-/-^). Bone marrow cells were cultured in the presence of M-CSF (20 ng/mL) (PeproTech, USA) for six days to generate the bone marrow-derived macrophages (BMDMs). BMDMs were cultured in DMEM (Hyclone, US) containing 10% fetal bovine serum (FBS) (Serana, AUS), 1% penicillin/streptomycin (Beyotine, China) in a humidified atmosphere with 5% CO_2_ at 37°C. Thereafter, BMDMs were stimulated by rCsHscB (5~40 μg/ml) or production of *E*. *coli* transfected by pET-28 control vectors for 24h, and supernatants were obtained for determining the concentration of IL-10 using ELISA. For ERK1/2 inhibitor assay, PD98059 (1μM) (Sigma, US) was pre-incubated with cells for 2 h, BMDMs from WT or Tlr2-/- mice were stimulated by rCsHscB (20 μg/ml) or Pam_3_CSK_4_ (200 ng/ml) (Invivogen, US) and supernatants were used for ELISA.

### Western blotting analysis

Total cell lysates, liver homogenate, ESPs or rCsHscB were separated by 10% SDS-polyacrylamide gel electrophoresis (PAGE) and transferred onto Immobilon-P Transfer Membranes (PVDF) (Millipore, USA). For detection specific antibodies against to rCsHscB in vivo, sera from *C*. *sinensis*-infected and non-infected mice, as well as C. sinensis crude antigens immunizing sera as primary antibodies for 12 h at 4°C, and then horseradish peroxidase-conjugated secondary antibody (Beyotine, China) were incubated. For detection of MAPK signaling, the PVDFs were blocked with 5% non-fat-milk in PBS-Tween (PBS-T) and incubated with anti-His (ZSGB-Bio, China), anti-phospho ERK (CST, US), anti-phospho p38 (CST, US), anti-TLR2 (CST, US), anti-rCsHscB, anti-β-actin (Beyotine, China), and horseradish peroxidase-conjugated secondary antibody (Beyotine, China). The PVDFs were visualized by ECL exposure to X-ray film. Densitometry analyses were performed by Image Lab software.

### Flow cytometry

Following stimulation, RAW264.7 were stained with TLR2 (eFlour 660), CD80 (PE), CD86 (eFlour 450), major histocompatibility complex class II (FITC), CD206 (PE/Cy7), CD11b (APC-Cy7). BMDMs were stained with TLR2 (eFlour 660), CD11b (APC-Cy7), F4/80 (Percp-Cy5.5). Antibodies were purchased from BD Pharmigen (US). Samples were analyzed with FlowJo software.

### ELISA

Supernatants from RAW264.7 or BMMs cultures were analyzed using commercially available ELISA kits for IL-10, IL- 6, and TNF-α (all from eBioscience, San Diego, CA, US).

### Pull-down assay

The cells were stimulated by supernatant of lysate from *E*. *Coli* transfecting with Vector controls (pET-28, His-tagged control), pET-28a-CsHscB vectors (pET-28-CsHscB, unpurified), purified rCsHscB-stimulated cells, binding buffer and medium for 24 h, subsequently, the cells from each group were lysed for further use. The rCsHscB were incubated with Ni-NTA beads (QIAGEN, GER) for 12h at 4°C after the agaroses were balanced with binding buffer at 4 times in 4°C. rCsHscB immobilized on bead were incubated with total cell lysates (RAW264.7) for 12h at 4°C. The supernatant was discarded after centrifuged at 2500 rpm for 5 minutes at4°C. The bead-bound proteins were subjected to 10% SDS-PAGE and then transferred electrophoretically to PVDF membranes. The membranes were incubated with anti-His antibody or anti-TLR2 antibody, followed by horseradish peroxidase-conjugated secondary antibody (Beyotine, China). The PVDFs were visualized by ECL exposure to X-ray film.

### Statistical analysis

All data were expressed as the mean ±standard error of the means (SEM). One-way ANOVA was used to analyze the significance of the differences between groups, followed by Tukey’s test using SPSS 13.0. For all tests, *P*<0.05 was considered statistically significant.

## Results

### Identification, characterization, and immunogenicity of recombinant *C*. *sinensis* HscB

**Fig 1:**
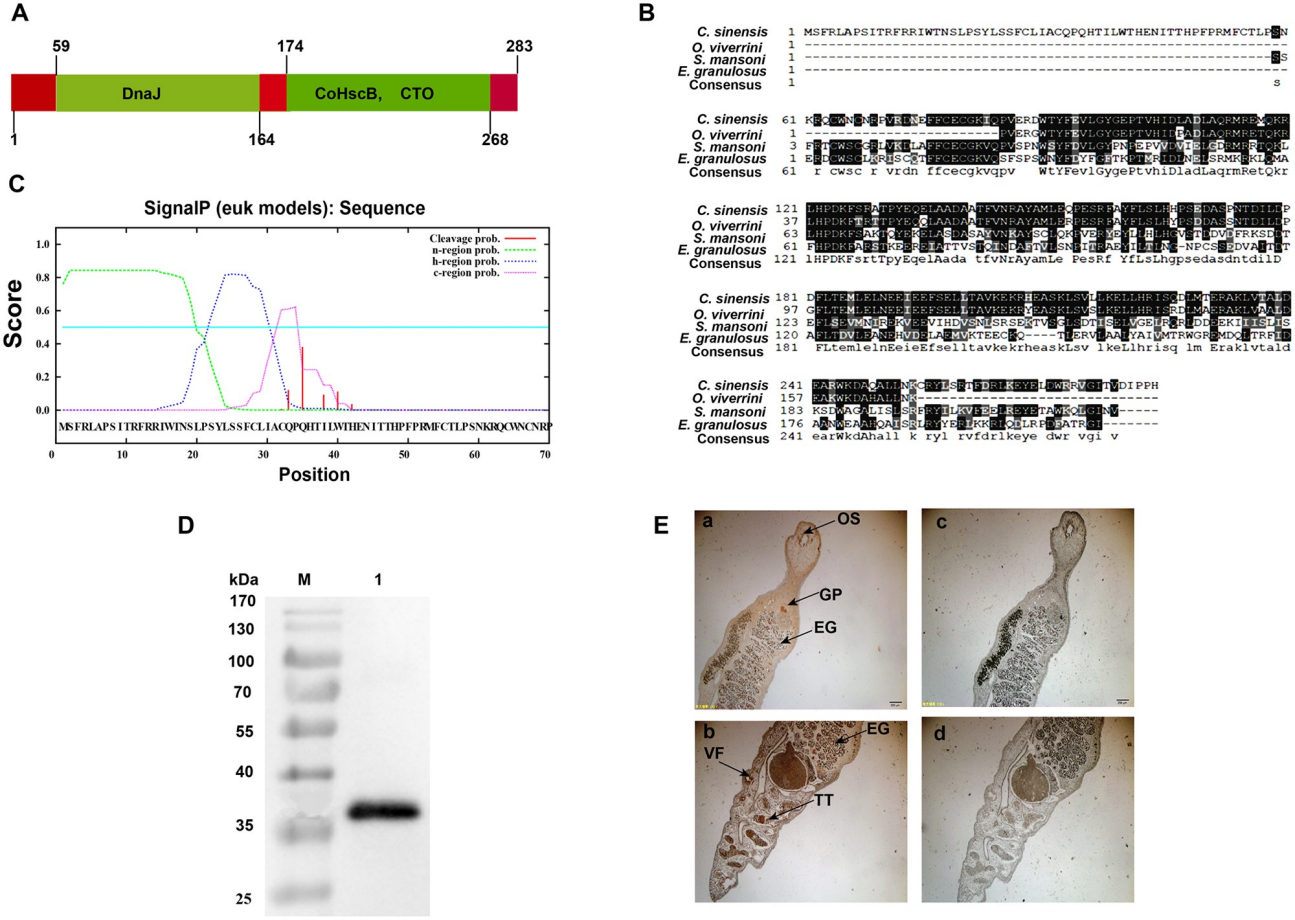
Identification, characterization, and immunogenicity of *Clonorchis sinensis*HscB. **(A)** Secondary structure of CsHscB including three domains: DnaJ, Co-chaperone HscB (CoHscB), and C-terminal oligomerisation (CTO). **(B)** Multiple protein sequences alignment of CsHscB among different species of the helminthes. (**C**) Signal peptide identification of CsHscB using SignalP server 3.0. **(D)** Western-blot analysis of purified rCsHscB using an anti-His antibody. **(E)** Expression and distribution of rCsHscB in the *C*. *sinensis* adult worms using IHC. a~b:rCsHscB on the worm bodies were detected using the primary antibody of rCsHscB; c~d: rCsHscB on the worm bodies were detected using IgG isotype as the primary antibody for controls. Arrows indicate the expression of rCsHscB mainly on the oral sucker (OS), vitelline follicles (VF), genital pore (GP), testis (TT), and eggs (EG). Scale bars, 20 μm.

As most TLR2 agonists or ligands have been reported as lipoproteins or lipopeptides [19], to identify potential agonist of TLR2 sourced from *C*. *sinensis*, we collected all the amino acid sequences encoding *C*. *sinensis* proteins from the proteome data (http://www.ncbi.nlm.nih.gov/bioproject/PRJDA72781) and then putative lipoproteins from *C*. *sinensis* proteome were screened and predicted using a combination of DOLOP, lipoP and Lipo database as previously described [9]. We ultimately identified a lipoprotein named molecular chaperone HscB (CsHscB), which had 283 amino acids with three domains as followed: DnaJ, Co-chaperone HscB (COHscB) and C-terminal oligomerization (CTO) (Fig 1A). Alignment of amino acid sequences analysis showed that the sequences of *C*. *sinensis* HscB had more than 90% similarities to *Opisthorchis viverrini* hypothetical protein (XP_009168973.1), but only had 40.91% similarities to the putative co-chaperone protein HscB from *Schistosoma mansoni* and 33.64% to the co-chaperone HscB from *Echinococcus granulosus* (Fig 1B). The candidate lipoproteins were further selected for the prediction of N-terminal signal peptide using SignaIP server 3.0 (http://www.cbs.dtu.dk/services/SignalP-3.0/) by a hidden Markov model (HMM) [20]. CsHscB had a signal peptide (the probability was 0.759, Fig 1C) and the predicted cleavage sites were at between N-terminal 34 and 35 sites (Fig 1C).

However, it was very difficult to isolate and purify CsHscB directly from the worms due to the lack of sufficient background information as well as the low yield. We, therefore, used a recombinant CsHscB (rCsHscB) that was routinely expressed by *E*. *coli* (Ec). rCsHscB was purified by nickel-affinity and ion-exchange chromatography, and the purified rCsHscB was assessed by western blot (Fig 1D). The molecular weight of rCsHscB including a 6×his tag was approximate 36 kDa (Fig 1D). Furthermore, we prepared the specific rCsHscB antibody to examine the expression and distribution of CsHscB in the worm body using immunohistochemistry (IHC). IHC data showed that CsHscB mainly expressed on the oral sucker (OS), genital pore (GP), vitelline gland (VF), ovary (OV), testis (TT) and eggs (EG) (Fig 1E). It could be detected by the sera from *C*. *sinensis*-infected mice as well as *C*. *sinensis* crude antigen- immunized mice, suggesting that rCsHscB was recognized by a pool of antibodies induced by *C*. *sinensis* and crude antigen as well. It is also suggested that CsHscB naturally existing in *C*. *sinensis*- infection mice and worm’s crude antigens can trigger host immune responses (S1A Fig). We also determined whether the CsHscB also was present ESPs of *C*. *sinensis* using western-blot or not, however, it could not be detected in ESPs (8 μg for western-blot), suggesting that CsHscB maybe not present in the ESPs of *C*. *sinensis* (S1B Fig).

### rCsHscB induces the activation of macrophage and cytokine production

**Fig 2:**
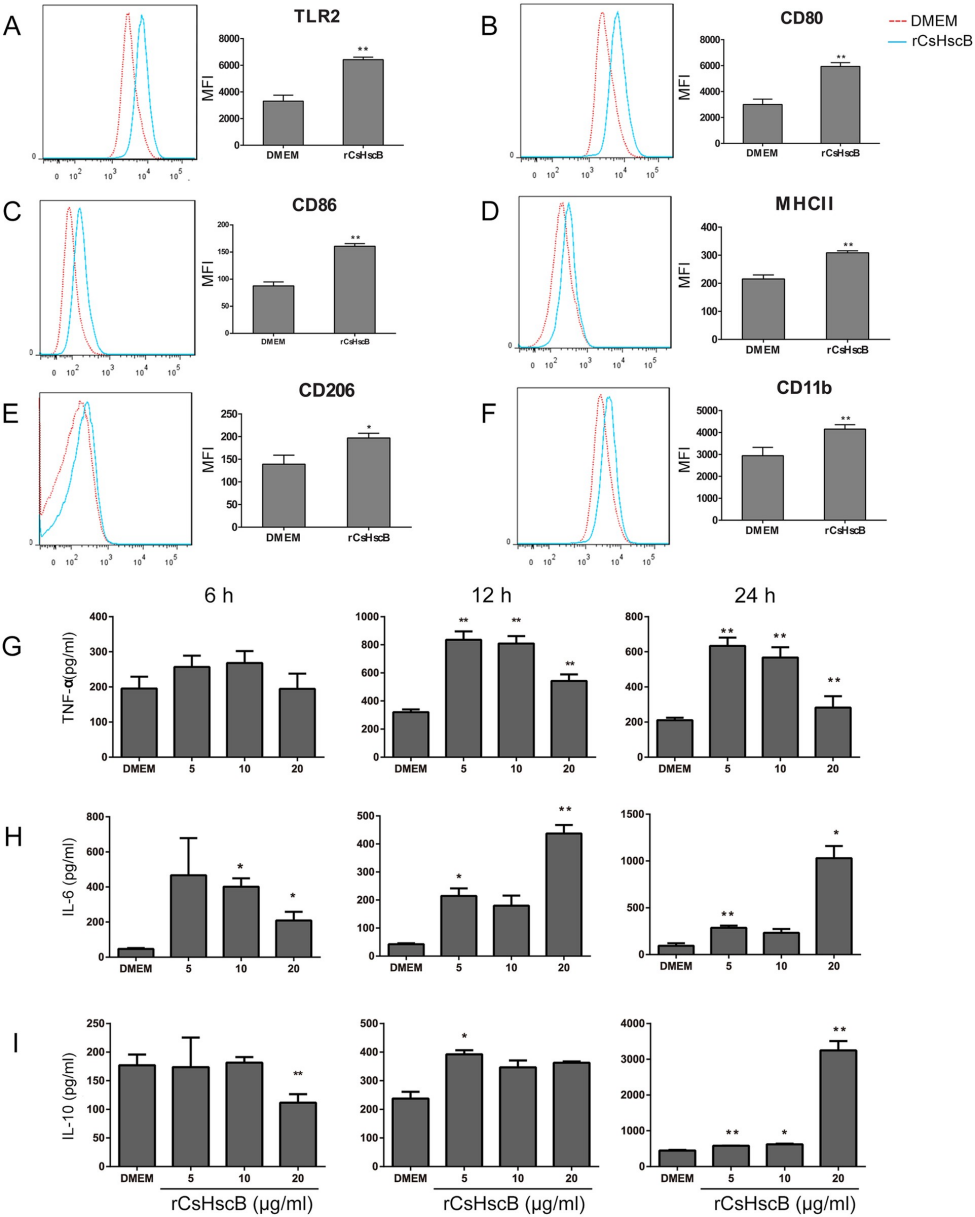
rCsHscB induces the activation of macrophage and cytokine production. (A~F) rCsHscB increased the expression of macrophage activation markers by flow cytometry. (G~I) productions of TNF-α (G), IL-6 (H), and IL-10 (I) were assayed for ELISA in the macrophage stimulated by indicated concentrations of rCsHscB for different time courses. Quantitative data are the mean±SE of three independent experiments, and all data shown are representative of at least three independent experiments (n = 4~5). *P< 0.05, **P< 0.01, and ***P< 0.001, stimulated cells *versus* those cultured in medium alone.

To highlight the role of rCsHscB in the activation of innate immune cells, we used various concentrations of rCsHscB to stimulate macrophage cell line-RAW 264.7 at different time-points. Firstly, we tested the toxicity of rCsHscB on macrophages, lactate dehydrogenase (LDH) test showed that up to 20 μg /ml the rCsHscB protein did not display any cellular toxicity against macrophages (S2 Fig). Furthermore, endotoxin (LPS) in the purified rCsHscB was removed by Endotoxin Erasol Solution (Tiandz, Beijing, China) to exclude any potential effects of LPS produced during preparation of rCsHscB. The concentration of endotoxin was detected by Limulus Amebocyte Lysate (LAL) and rCsHscB solution with less than 0.1 EU/ml of endotoxin should be further studied. For the assessment of the activation of macrophages, we detected activation markers of macrophages upon stimulation using flow cytometry. The data showed that stimulation of macrophage with rCsHscB (20 μg/ml) for 24 h augmented the surface expression of activation markers such as TLR2, CD80, CD86, MHCII, CD206 and CD11b (Fig 2A~2F). We also detected these cytokines with various concentrations (5~20 μg/ml) of rCsHscB at different time courses, and the results showed that macrophages stimulated by 5~20 μg/ml rCsHscB for 12 h produced high levels of TNF-α (Fig 2G). Also, rCsHscB with a concentration of 5~10 μg/ml but not 20 μg/ml for 24 h still induced a robust secretion of TNF-α by macrophage (~3 times greater than DMEM-stimulated cells, Fig 2G). The cells also produced high levels of IL-6 under the stimulation with 5~20 μg/ml rCsHscB for 12 h or 24 h, compared with medium-stimulated cells (Fig 2H, *P*<0.05). Concerning IL-10, cells stimulated with 5~20 μg/ml rCsHscB for 12 h or 24 h could produce a robust increase of IL-10, of note, the secretion of IL-10 in macrophage stimulated by 20 μg/ml rCsHscB for 24 h was more than 10 times greater than that of DMEM-stimulated cells (Fig 2I). We also tested the levels of IL-4 and IL-12 produced by macrophage stimulated by rCsHscB, but the data showed that the stimulation of macrophage by rCsHscB didn’t induce an increase in IL-4 and IL-12 production (S3A and S3B Fig).

To exclude any potential effects of endotoxin and other potential component produced during preparation of rCsHscB on the activation of macrophage, we also compared the productions of *E*. *Coli* induced by pET-28a vector with or without CsHscB open reading frame (pET-28a-CsHscB or control vector), the production induced by control vector could not stimulate macrophage to secrete high levels of IL-10 and TNF-α (S4A and S4B Fig). However, it seems that the cells that were stimulated by the production expressed by control vector also secreted a higher level of IL-6, compared with DMEM stimulated cells, although the level of IL-6 was still lower than that of pET-28a-CsHscB-induced cells, suggesting that the increased secretion of IL-6 maybe not exclusively induced by rCsHscB (S4C Fig). Taken together, these data demonstrate that rCsHscB triggers the activation of macrophage and induces a robust cytokine production by the macrophage.

### rCsHscB is an unidentified agonist for TLR2 to induce immune responses of macrophage

**Fig 3:**
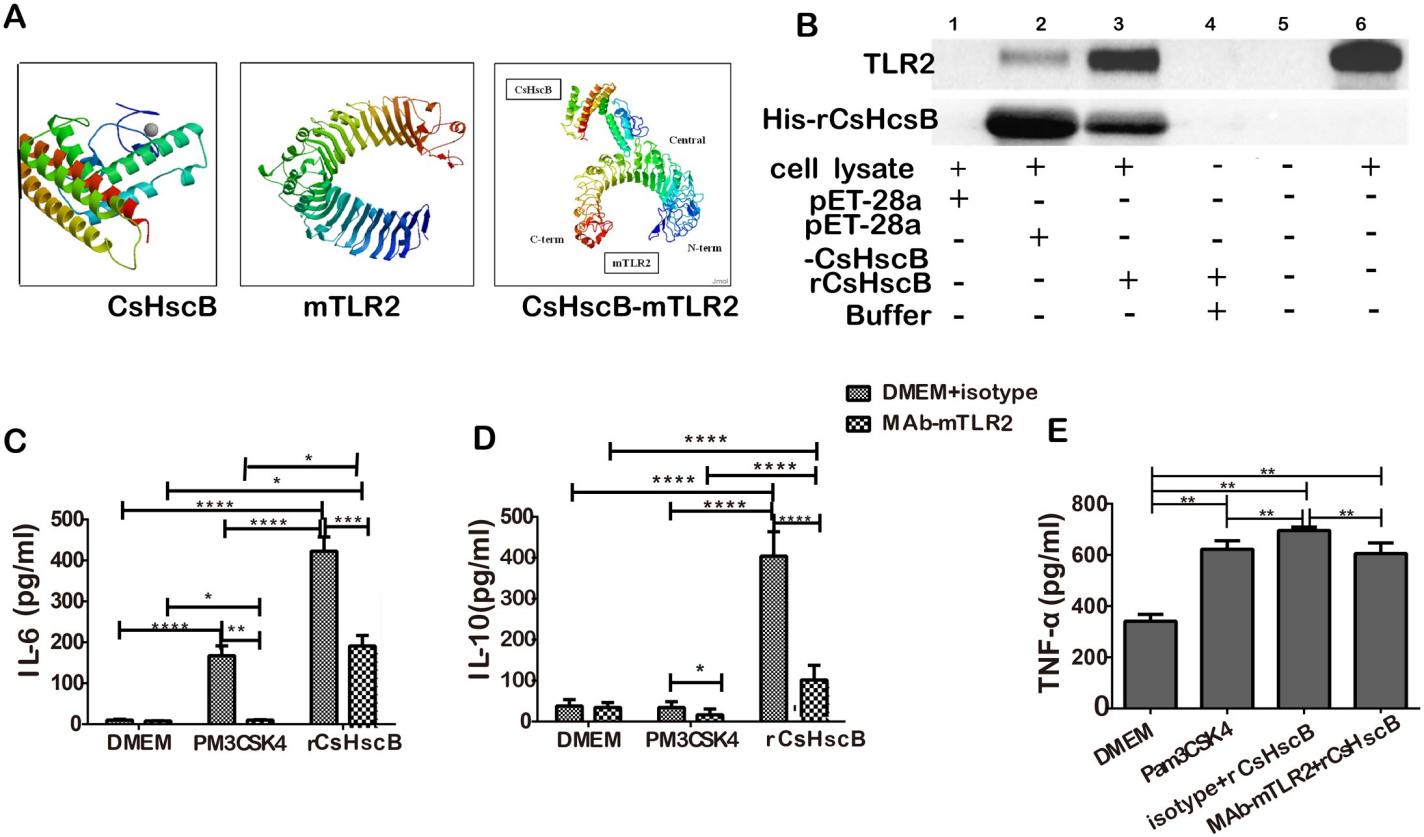
rCsHscB is a novel agonist for TLR2 to induce immune responses of macrophage. **(A)** Molecular docking analysis of binding TLR2 with CsHscB. **(B)** Pull-down assay analysis of the interaction of TLR2 and rCsHscB. The cells stimulated by supernatant of lysate from *E*. *Coli* transfecting with Vector controls (pET-28, His-tagged control, Lane 1), pET-28a-CsHscB vectors (pET-28-CsHscB, unpurified, Lane 2), purified rCsHscB-stimulated cells (Lane 3), binding buffer (Lane 4) and medium (Lane 6) for 24 h, the cells (from line 1~ line 5) were lysed and incubated with rCsHscB immobilized on Ni-NTA beads; bead-bound proteins (from line 1~ line 5) and cell lysates (line 6 without rCsHscB immobilized on Ni-NTA beads) were loaded onto a gel for immunoblotting for both TLR2 and rCsHscB. Lane 5 represents negative control for pull-down assay; line 6 represents positive control for TLR2. **(C~E)** The production of IL-6 **(C)**, IL-10 **(D),** and TNF-α **(E)** were hindered in rCsHscB-stimulated cells when TLR2 was blocked by a neutralizing antibody of TLR2. PM_3_CSK_4_ was used as TLR2 ligand for positive controls. Quantitative data are representative of mean ± SEM of at least three independent experiments (n = 4).*P < 0.05, **P< 0.01, and ***P< 0.001 compared with indicated group.

As the lipoproteins or lipopeptides have been reported as TLR2 agonist or ligand [19], we next tested whether rCsHscB as an agonist for TLR2 can promote the activation of macrophage or not. Firstly, we performed *in silico* molecular docking using the crystal structure of the extracellular domain (ECD) of mouse TLR2 and modeled the 3D structure of rCsHscB by homology modeling. Molecular docking showed that CsHscB could bind with TLR2 at its leucine-rich region (LRR) 11~15 sites of ECD (Fig 3A). To further ascertain whether rCsHscB physically interacts with the TLR2 molecule or not, we performed a pull-down assay using whole-cell extracts from RAW247.6 cells stimulated by rCsHscB. The cell extracts were incubated with rCsHscB immobilized on Ni-NTA beads. TLR2 pull-down assay revealed that supernatant of lysate from *E*. *Coli* transfecting with pET-28a-CsHscB vectors as well as purified rCsHscB proteins could pull-down TLR2 molecule as showed by western-blot (Fig 3B line 2 and line 3), demonstrating that TLR2 and rCsHscB could physically interact. However, without rCsHscB (control vectors for example), no bands were observed on the gel of western-blot, suggesting that, there are no interactions between TLR2 and other molecules except rCsHscB (Fig 3B line 1 and line 4). Collectively, these data suggested that rCsHscB interacts specifically and predominantly with TLR2.

To test whether rCsHscB can induce the cytokines production in a TLR2-dependent manner or not, TLR2 was blocked by pretreating RAW 264.7 cells with anti-TLR2 antibody (T2.5) for 2 h prior to the addition of rCsHscB. The secretion of IL-6 and IL-10 induced by rCsHscB were almost abolished following the addition of TLR2 blocking antibodies to the medium (Fig 3C and 3D). Similarly, rCsHscB-induced TNF-α was also significantly abolished due to the presence of the TLR2 blocking antibody (Fig 3E).

### rCsHscB-induced IL-10 production partly depends on phosphorylation of ERK1/2 but not p38 in RAW 264.7 cells

**Fig 4:**
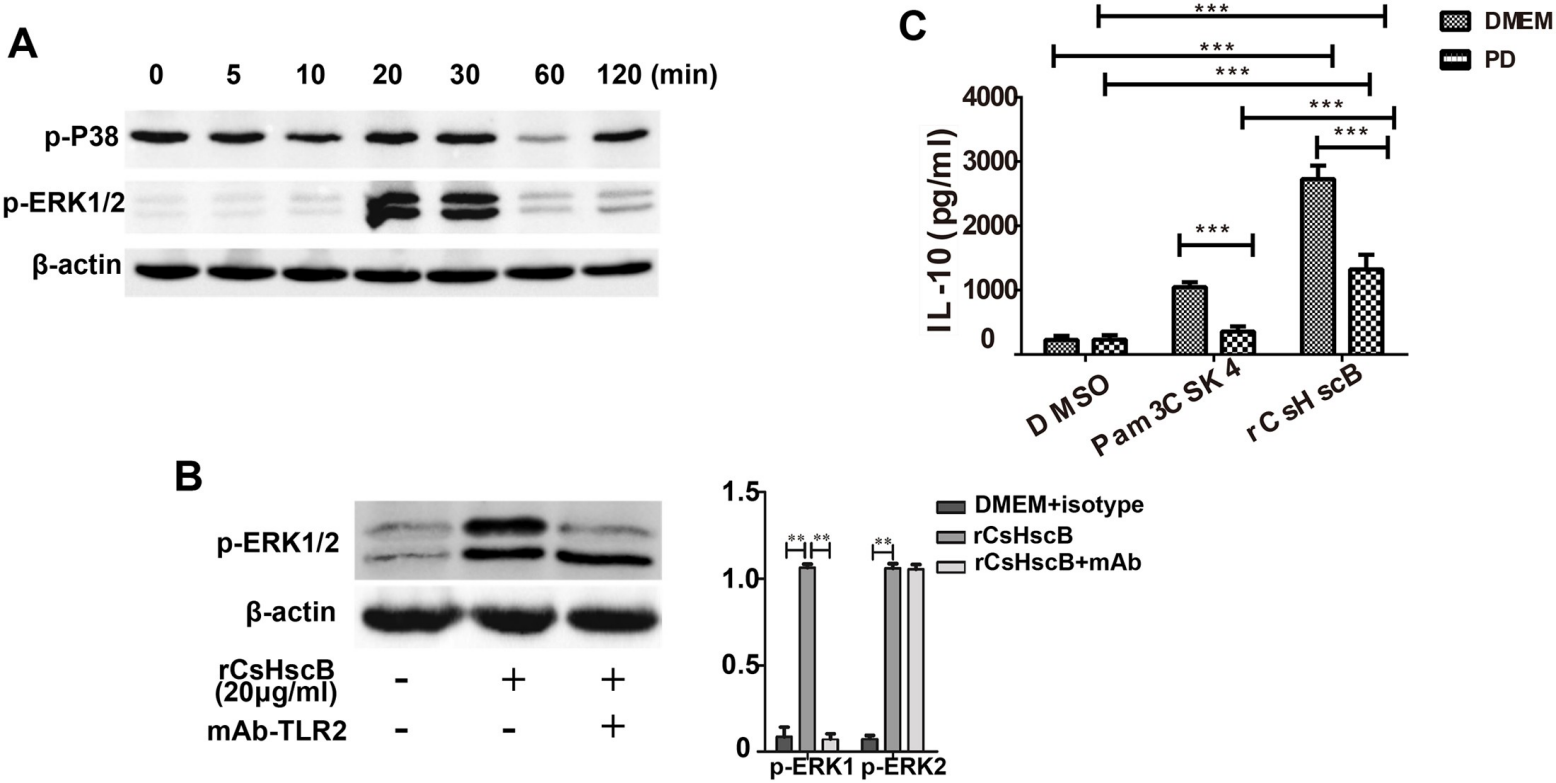
rCsHscB-induced IL-10 production partly depends on TLR2-mediated phosphorylation of ERK1/2 in RAW 264.7 cells. **(A)** ERK1/2 but not p38 MAPK was activated in rCsHscB-stimulated RAW 264.7 macrophage for 20~120 min detected by western-blot. **(B)** phosphorylation of ERK1 but not ERK2 was attenuated in rCsHscB-stimulated cells when TLR2 was blocked by neutralizing antibody determined by western-blot. **(C)** ELISA analysis of IL-10 production in rCsHscB-stimulated RAW 264.7 cells the presence or absence of ERK1/2 inhibitor (PD98059). Quantitative data are representative of mean ± SEM of at least three independent experiments (n = 4). *P< 0.05, **P< 0.01, and ***P< 0.001 compared with indicated group.

As shown by our and others’, IL-10 might play a regulatory role in immune responses during *C*. *sinensis* infection [18, 21–23], which highlights the significance of rCsHscB induced a strong IL-10 production in macrophage in our study. It was therefore necessary to further study the mechanisms that IL-10 induced by rCsHscB. For this sense, to determine which downstream molecules mediated by TLR2 are responsible to the robust rCsHscB-induced IL-10 production by macrophage, we screened the activation of transcription factors nuclear factor-κB (NF-κB), p38 mitogen-activated protein kinase and ERK1/2 in RAW264.7 cells using optimal concentrations (20 μg/ml) of rCsHscB during various time courses, western blot showed that rCsHscB induced a strong phosphorylation of ERK1/2 after 20~30 min and then the levels of phosphorylation of ERK1/2 was attenuated during 60 min~120 min following stimulation with rCsHscB (Fig 4A). Surprisingly, there was neither obvious activation of NF-κB nor p38 during these time courses (Fig 4A). Furthermore, we also examined whether the rCsHscB-induced phosphorylation of ERK1/2 was mediated by TLR2 signaling. Western blot showed that phosphorylation of ERK1 but not ERK2 was solely abolished following the addition of TLR2 blocking antibodies to the cultures, compared with isotype-matched control (Fig 4B). Also, we used a specific inhibitor for ERK1/2 (PD98059) to examine whether rCsHscB-induced cytokines were mediated by ERK signaling pathway or not. The RAW264.7 cells were pretreated with 10 μM PD98059 for 2 h, and then stimulated by 20 μg/ml rCsHscB for 24 h, the supernatants were collected for IL-10 detection. The data showed that the level of IL-10 was significantly decreased when ERK1/2 was inhibited by PD98059 in macrophage that was stimulated by rCsHscB for 24 h (Fig 4C, *P*<0.001, ~50% decreased).

### rCsHscB-induced IL-10 production depends on TLR2-mediated ERK1/2 signaling in bone marrow-derived macrophage

**Fig 5:**
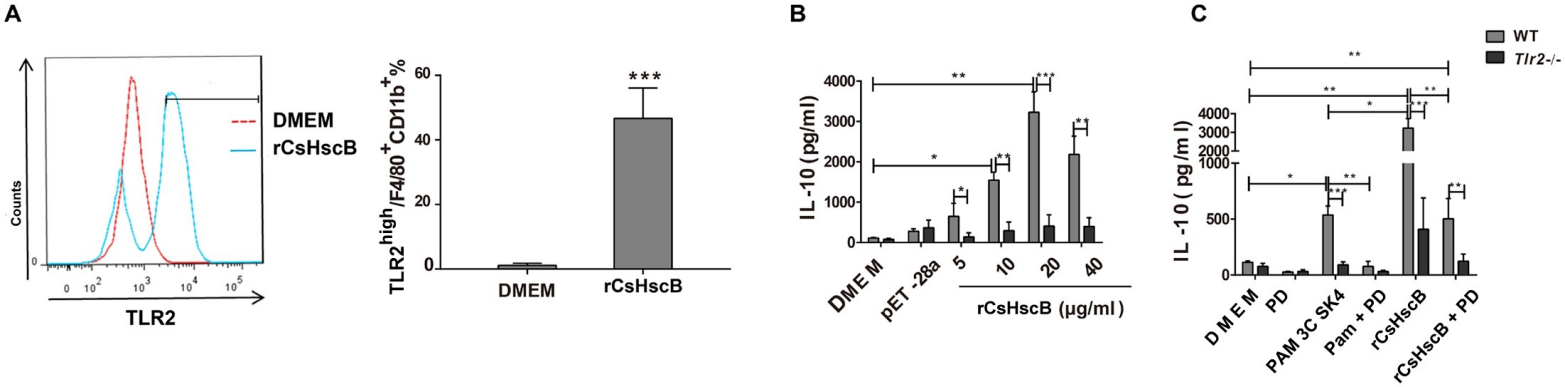
rCsHscB-induced IL-10 production depends on TLR2-mediated ERK1/2 signaling in bone marrow-derived macrophage. **(A)** Flow cytometry analysis of TLR2^high^ F4/80^+^CD11b^+^ in DMEM stimulated and rCsHscB-stimulated BMDMs from TLR2 wild type mice. **(B)** ELISA analysis of IL-10 production in BMDMs cells from TLR2 wild type and *Tlr2*^-/-^ mice stimulated with various concentrations of rCsHscB as indicated. DMEM as negative control. pET-28 group represents supernatant of lysate from *E*. *Coli* transfecting with pET-28a empty vector. **(C)**ELISA analysis of IL-10 production in BMDMs cells from TLR2 wild type and *Tlr2*^-/-^ mice stimulated with rCsHscB (20 μg/ml) with or without of ERK1/2 inhibitor (PD98059). PM_3_CSK_4_ was used as TLR2 ligand for positive controls. Quantitative data are representative of mean ± SEM of at least three independent experiments (n = 4). *P< 0.05, **P< 0.01, and ***P < 0.001 compared with indicated group.

To ascertain the roles of TLR2-regulated ERK1/2 signaling in rCsHscB-induced IL-10 in macrophage, we induced bone marrow-derived macrophage (BMDM) from *Tlr2* wild type and *Tlr2*^-/-^ mice. The production of TNF-alpha and IL-6 can not be triggered in the homozygotes of *Tlr2*^-/-^ with C57BL/6 background [24]. Similar to our previous data, rCsHscB could potently induce a strong TLR2 expression on the surface of BMDM sourced from wild type mice (Fig 5A, almost 2 fold changes) and the levels of IL-10 were significantly increased when BMDM cells from *Tlr2* wild-type mice were stimulated with various concentration of rCsHscB (5~40 μg/ml), compared with medium or the production of *E*. *coli* transfected by the empty vector (Fig 5B, *P*<0.001). Furthermore, the production of IL-10 reached a peak at the concentration of 20 μg/ml (almost 6 times increase). However, rCsHscB-induced IL-10 production in BMDM from TLR2 knockout mice was nearly abrogated (Fig 5C).

To verify whether rCsHscB-induced IL-10 production was dependent on the TLR2 mediated ERK1/2 signaling pathway, we used an inhibitor of ERK1/2 pretreated BMDM cells sourced from TLR2 wild type and TLR2 knockout mice and then stimulated by 20μg/ml rCsHscB for 24 h, IL-10 production in the culture were detected using ELISA. As previously demonstrated, the secretion of IL-10 in BMDM cells from TLR2 knockout mice was almost abolished when BMDM cells were stimulated by rCsHscB for 24 h (Fig 5C). For TLR2 wild type BMDM cells, the results showed that there was a significant decrease of IL-10 production in the BMDM cells with pretreatment of PD98059, compared with the cells pretreated by DMSO (the vehicle for PD98059). Furthermore, the data also demonstrated that the production of IL-10 was remarkably decreased (~4 times decreased, Fig 5C) in rCsHscB stimulated BMDM cells derived from *Tlr2*^-/-^ mice compared with that from *Tlr2* wild type mice. Collectively, our data demonstrated that rCsHscB induced IL-10 production in macrophage depends on the activation of TLR2-depended ERK1/2 signaling.

### rCsHscB could induce IL-10 in the liver of mice dependently by TLR2 mediated signaling pathway

**Fig 6:**
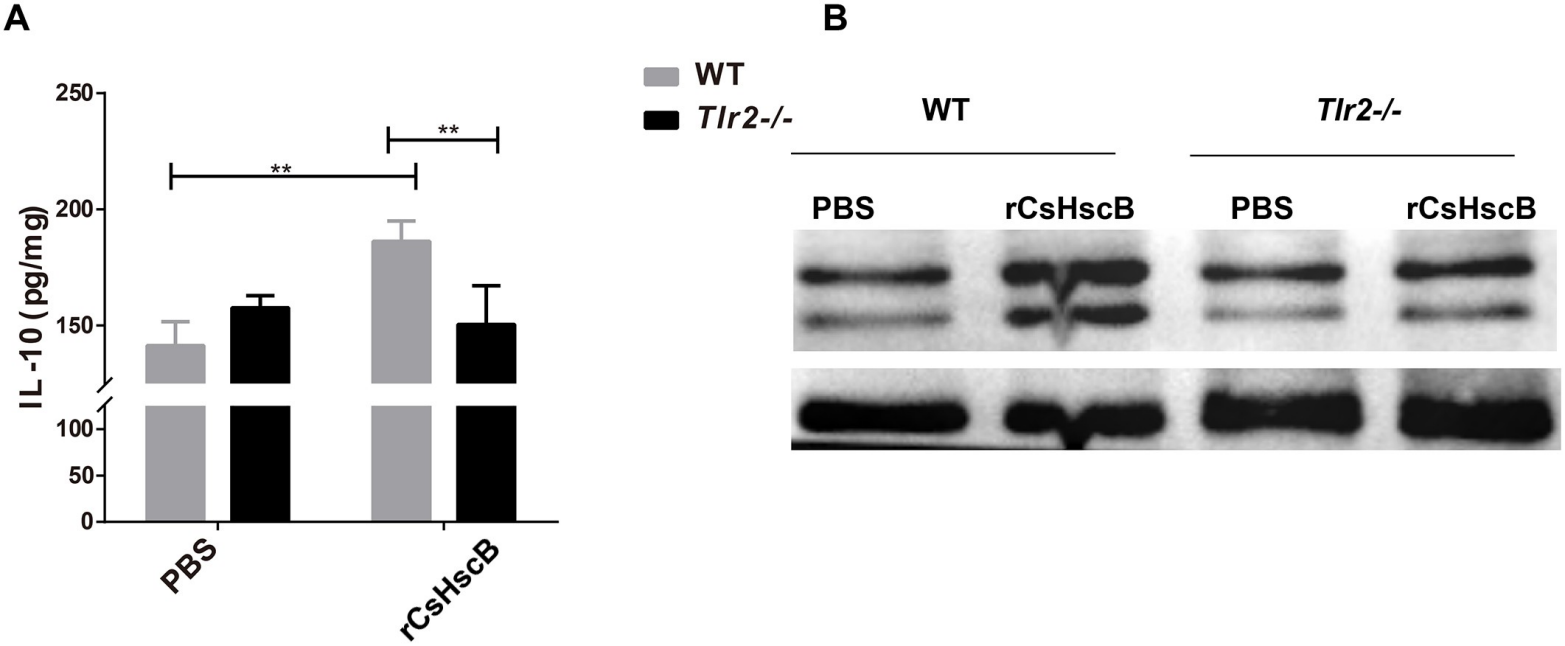
rCsHscB increased IL-10 production in the liver of mice via TLR2/ERK1/2 signaling pathway. The mice of TLR2 wild type and TLR2 KO mice were administrated with rCsHscB or PBS for 24 h *i*. *v*., and the mice were sacrificed and the livers from each mouse were collected for IL-10 and p-ERK1/2 detection. **(A)** IL-10 production in supernatant of hepatic homogenate from each group was determined by ELISA. **(B)** Phosphorylation of ERK1/2 was attenuated in TLR2 KO mice that were received rCsHscB determined by western-blot. Quantitative data are representative of mean ± SEM of at least three independent experiments (n = 5). **P< 0.01, compared with the indicated group.

To test whether rCsHscB could induce IL-10 production mediated by TLR2/ERK1/2 signaling pathway *in vivo* or not, the mice with or without *Tlr2* both received rCsHscB (5 mg/kg body weight) or PBS by *i*. *v*. for 24 h, the levels of IL-10 in the hepatic homogenate were determined. The data showed that rCsHscB induced a strong production of IL-10 in the liver of mice, compared with the PBS group (Fig 6A, *P*<0.01). However, IL-10 production in the liver from *Tlr2*^-/-^ mice were significantly lower than those in *Tlr2* wild type mice when they were both stimulated with the same dose of rCsHscB (Fig 6A, *P*<0.01), but there was no any statistic difference in IL-10 in the supernatant of hepatic homogenate in rCsHscB *Tlr2*^-/-^mice and those from PBS treated *Tlr2*^-/-^ mice (Fig 6A, *P*>0.05), suggesting that rCsHscB also induced IL-10 production in a TLR2 dependent manner *in vivo*. Furthermore, we also found that the phosphorylation of ERK1/2 in livers of *Tlr2*^-/-^ mice was also attenuated, compared with *Tlr2* wild type mice following administration of the same dose of rCsHscB (Fig 6B). Collectively, these data demonstrated that rCsHscB could induce IL-10 production mediated by the TLR2/ERK1/2 signaling pathway.

## Discussion

*C*. *sinensis* has evolved complex mechanisms for resistance to immune responses. Zhao et al demonstrated that total protein from *C*. *sinensis* inhibited Th1 immune responses by the activation of mannose receptor (MR), but not TLR2 or TLR4 to induce Th2-skewed response [15]. Our previous study showed that during the secretion of ESPs by *C*. *sinensis*, TLR4 plays a regulatory role in the induction of type I-relative cytokines (such as IFN-γ, IL-12, IL-6, TNF-α) [14]. However, the evidence suggests that the complex mechanisms for host-parasites interaction during *C*. *sinensis* infection are still poorly understood.

Many lipoproteins or lipo-peptide have been reported to display TLR2 ligands or agonists activities such as *Mycobacterium tuberculosis* (Mtb) LprG [5], MtbLprA [25], *schistosomal* lyso-PS [8] and filarial Diacyl WoLP [9]. Thus, to identify the potential TLR2 agonist sourced from *C*. *sinensis*, we screened the *C*. *sinensis* proteome data and predicted the potential lipoproteins using bioinformatics analysis. A lipoprotein from the family Co-chaperone Hsc20 (CsHscB) was ultimately selected for further study. However, it is very difficult to purify CsHscB directly from the worms due to the lack of sufficient background information as well as the low yield. We, therefore, used a recombinant CsHscB by *E*. *coli*, which was also recognized by sera of *C*. *sinensis* infect-mice, suggesting that rCsHscB remains the immunogenicity of *C*. *sinensis* rCsHscB. It was found that rCsHscB with a concentration of 5~20 μg/ml could induce a strong production of IL-10 by macrophage in a dose-dependent manner. Similarly, it has been also demonstrated that recombinant PPE18 from M. tuberculosis or Pam_3_CSK_4_ known as the TLR2 ligands also triggers the activation of macrophages and the production of IL-10 in a dosed manner by specifical interaction with TLR2 [26, 27]. Therefore, 20 μg/ml of rCsHscB was used as the optimized concentration for further study.

Pull-down assay is a useful approach to verify the protein-protein interaction *in vitro*. Using this assay, Chen et al. demonstrated that recombinant MPT83 derived from *M*. *tuberculosis* interacts specifically with TLR2 to promote the function of macrophage [28]. Our data suggested that rCsHscB sourced from *C*. *sinensis* might be acting as a TLR2 agonist plays a regulatory role in the immune responses to *C*. *sinensis* infection. However, the mechanisms by which TLR2 interacts with rCsHscB are not well known due to its complexity and further studies should be warranted. In our present study, we also found that the expression patterns of TNF-α, IL-6, and IL-10 secreted by macrophages were distinct using the different concentrations of rCsHscB at different time-courses (Fig 2G–2I), which reflects the functional differences of these mediators and the different mechanism of production [29]. For example, IL-6 as a lymphocyte stimulating factor was first produced to induce innate and adaptive immune responses in the early infection, but IL-10 is a regulator to alleviate hyper-inflammation for prevention tissues damage in the late infection [30, 31].

During chronic infection, parasite products trend to induce strong regulatory responses which may be in charge of balanced host-parasite interaction whereby the tissue damages were impeded and worms’ survival was favored. IL-10 has been known as one of the mechanisms that contribute to induced regulatory responses induced by helminth infection [32]. For example, the increased production of IL-10 is mainly responsible for the induction of CD4^+^ T cell hypo-responsiveness in the skin- draining lymph nodes after repeated exposure to *Schistosoma mansoni* larvae [33]. It is also evident that IL-10 sourced from CD4^+^CD25^-^ effector T cells impair IFN-γ production for the control of acute inflammation and myositis in the diaphragm caused by *Trichinella spiralis* as well [34, 35]. In respect of *C*. *sinensis*, it showed that augment IL-10 was triggered by dendritic cells treated by *C*. *sinensis* crude antigen [15, 36]. Furthermore, it’s found that IL-10 secreted by lymphocytes from FVB was significantly higher than by those of BALB/c mice, which suggests that IL-10 may contribute to the susceptibility of different strains mice [22, 37]. In our present study, rCsHscB interacted with TLR2 can potently induce IL-10 production in macrophage with various concentrations (5~20 μg/ml), which may account for mechanisms underlying the production of IL-10 driven by *C*. *sinensis* infection.

Macrophage is one of the important sources of IL-10 in responses to TLRs or other PRRs ligands. But the intrinsic mechanisms that tailored production of IL-10 are still poorly understood. MAPKs signaling pathways (such as ERK, p38, etc) have been suggested to be involved in the control of the production of IL-10 [38]. In our present study, we found that phosphorylated ERK but not p38 was activated in the responses to the stimulation of macrophage by rCsHscB for 20~30 min. Furthermore, the phosphorylated ERK1/2 was inhibited when the cells were pretreated by TLR2 blocking antibodies whereby the production of IL-10 was almost impaired, which suggests that the activation of ERK induced by rCsHscB in macrophage is mediated by TLR2. Interestingly, although the production of IL-10 in rCsHscB induced cells was significantly decreased when the cells were pretreated by PD98059, these amounts were still higher than DMEM controls. To confirm these data, we also used bone marrow-derived macrophage isolated from TLR2 wild and TLR2 knockout mice. Similar to our previous observation, the BMDM from TLR2 knockout mice impaired the secretion of IL-10 using the same amount of rCsHscB for the stimulation, compared with those from *Tlr2*^-/-^ mice, but IL-10 secreted by macrophages from TLR2 wild type mice after the stimulation with rCsHscB were partial depressed when ERK1/2 inhibitor was pretreated. These data suggested that, in addition to ERK, other signaling pathways may be also involved in the production of IL-10 in TLR2-dependent in macrophage-induced by rCsHscB. In addition, we also found that rCsHscB could induce the production of IL-10 via TLR2/ERK signaling pathway in mice following intraperitoneal injection with rCsHscB (5 mg/kg body-weight for 24 h), although there was a limitation that the concentration of rCsHscB used the study might be not associated with real *C* .*sinensis* infection and it also cannot be determined that the sources of CsHscB-induced IL-10 purely or partly due to an effect on macrophages.

In conclusion, in the present study, we identified CsHscB sourced from *C*. *sinensis* acting as a novel TLR2 agonist to induce potently activation of macrophage, our study also demonstrates that a robust IL-10 production by macrophage stimulated by rCsHscB is mediated by TLR2/ ERK1/2 signaling pathway, which may reveal a novel mechanism for host-parasites recognition during *C*. *sinensis* infection. The present study will contribute to a better understanding of the interaction between the worms and host cells. Besides, rCsHscB might be suggested in the development of novel therapeutic strategies with implications beyond parasitology due to its potential regulatory capacities of immune responses.

## Supporting information

S1 Fig CsHscB naturally existing in *C*. *sinensis*- infection mice and worm’s crude antigens can trigger host immune responses. **(A)** rCsHscB was recognized by pool of antibodies induced by *C*. *sinensis* and its crude antigen using Western-blot. Line 1: using sera from *C*. *sinensis*-infected mice 4 week post infection as a primary antibody; Line 2: using sera from non-infected mice as negative control. Line 3: using sera from *C*. *sinensis* crude antigen-boost mice as a primary antibody. (B) The detection of CsHscB in the ESPs using Western-blot. Line 1 (8 μg ESPs from C. sinensis), Line 2 (2 μg rCsHscB), Line 3 (12 μg) and Line 4 (24 μg rCsHscB) were loaded onto gel to subjected SDS-PAGE, and rabbit-sourced rCsHscB poly-antibodies were used as a primary antibody to detect rCsHscB using western-blot.
(TIF)

S2 Fig The toxicity of rCsHscB to macrophages. The activities of LDH in the medium of cultured cells stimulated by 5~20 μg/ml rCsHscB were determined by a using the commericial LDH colorimetric assay kit (n = 4).
(TIF)

S3 Fig The production of IL-4 and IL-12 by macrophage stimulated by rCsHscB. **(A)** The levels of IL-4 in the Raw 264.7 stimulated by various concentrations of rCsHscB for 6 h, 12 h, 24 h, respectively. **(B)** The levels of IL-12p70 were determined by ELISA using 5, 10, 20 μThe for 24 h.
(TIF)

S4 Fig The effects of endotoxin and other potential component produced during preparation of rCsHscB on the activation of macrophages. The production of IL-10 **(A)**, TNF **(B)** and IL-6 **(C)** in supernatants of Raw 264.7 cells stimulated by LPS (100 ng/ml, pET-28a empty (the production of *E*. *Coli* induced by pET-28a empty vector without CsHscB open reading frame), the elution buffer (containing unbound proteins in CsHscB solution during liquid chromatography) and the purified rCsHscB (20 μg/ml) for 24 h. Quantitative data are representative of mean ± SEM of at least three independent experiments. Compared with indicated group, * *P*<0.05, **P<0.01, ***P<0.001.
(TIF)
