# Supplementary figures and images for: Recombinant CsHscB of carcinogenic liver fluke Clonorchis sinensis induces IL-10 production by binding with TLR2
Source: PLoS Negl Trop Dis. 2020 Oct 12;14(10):e0008643. doi: 10.1371/journal.pntd.0008643 (PMC7549790; doi:10.1371/journal.pntd.0008643)

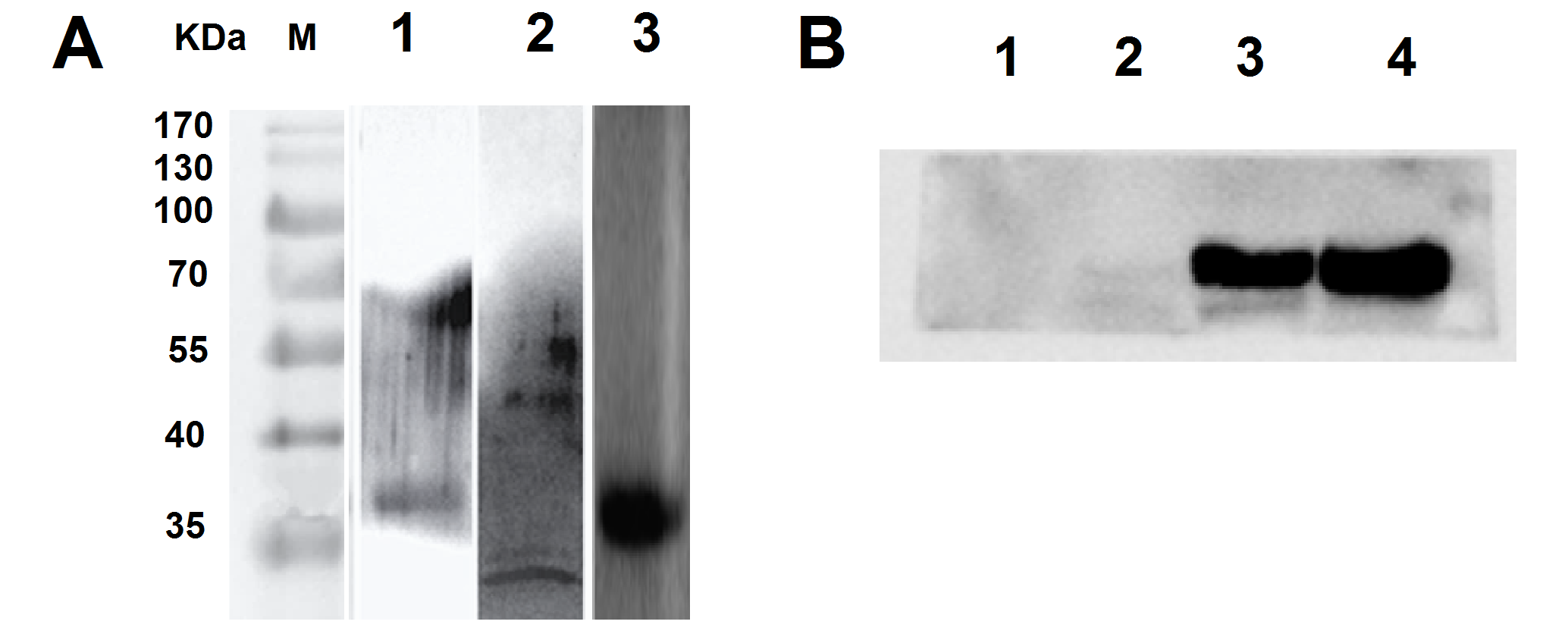

Supplement: S1 Fig — (A) rCsHscB was recognized by pool of antibodies induced by C. sinensis and its crude antigen using Western-blot. Line 1: using sera from C. sinensis-infected mice 4 week post infection as a primary antibody; Line 2: using sera from non-infected mice as negative control. Line 3: using sera from C. sinensis crude antigen-boost mice as a primary antibody. (B) The detection of CsHscB in the ESPs using Western-blot. Line 1 (8 μg ESPs from C. sinensis), Line 2 (2 μg rCsHscB), Line 3 (12 μg) and Line 4 (24 μg rCsHscB) were loaded onto gel to subjected SDS-PAGE, and rabbit-sourced rCsHscB poly-antibodies were used as a primary antibody to detect rCsHscB using western-blot. (TIF) [file pntd.0008643.s001.tif]

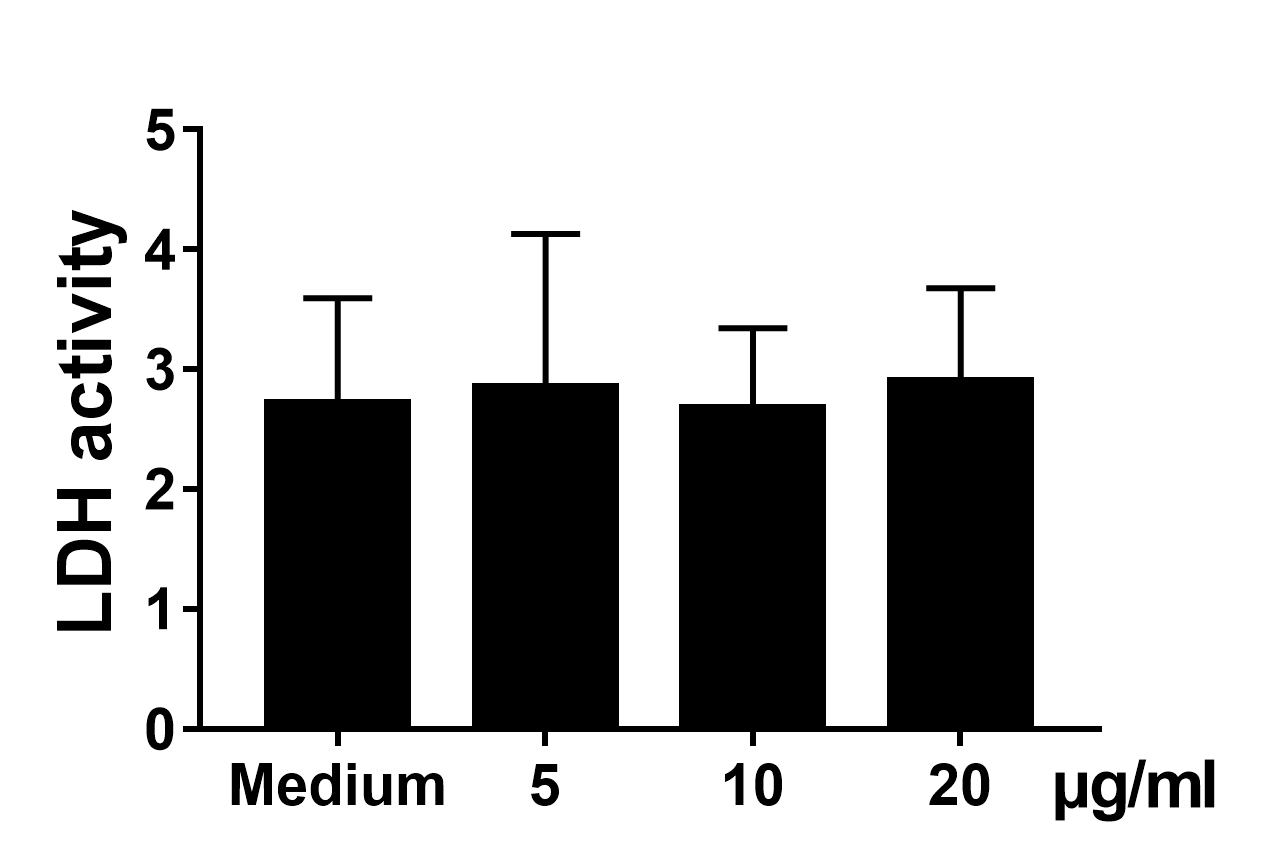

Supplement: S2 Fig — The activities of LDH in the medium of cultured cells stimulated by 5~20 μg/ml rCsHscB were determined by a using the commericial LDH colorimetric assay kit (n = 4). (TIF) [file pntd.0008643.s002.tif]

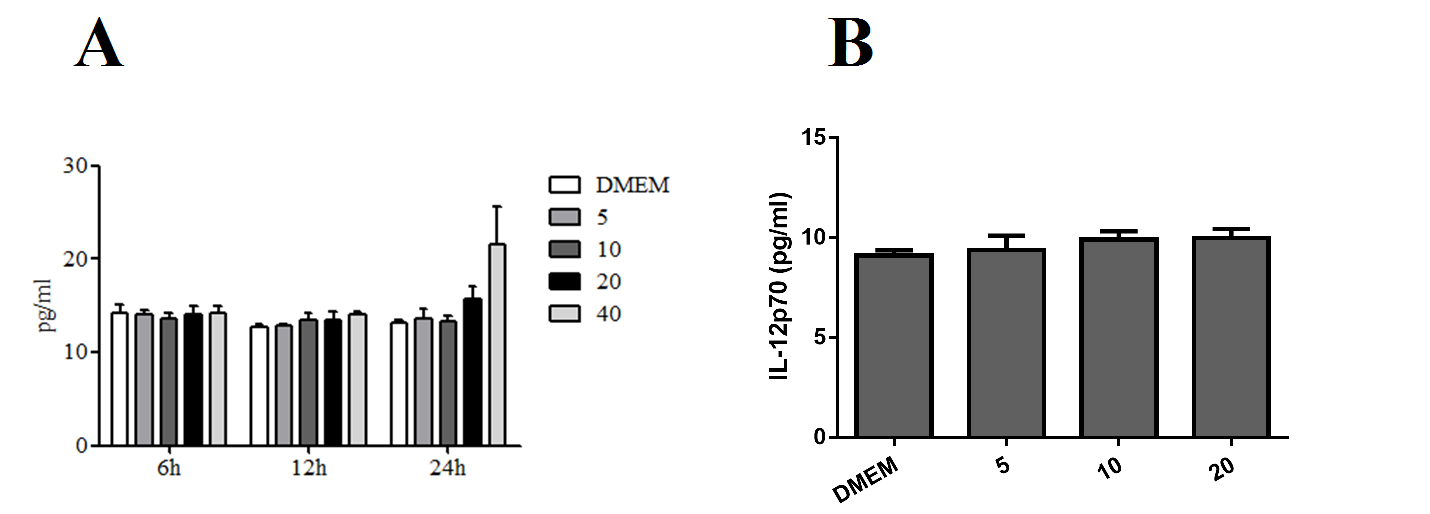

Supplement: S3 Fig — (A) The levels of IL-4 in the Raw 264.7 stimulated by various concentrations of rCsHscB for 6 h, 12 h, 24 h, respectively. (B) The levels of IL-12p70 were determined by ELISA using 5, 10, 20 μThe for 24 h. (TIF) [file pntd.0008643.s003.tif]

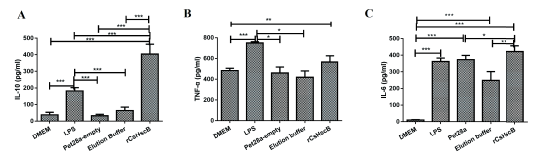

Supplement: S4 Fig — The production of IL-10 (A), TNF (B) and IL-6 (C) in supernatants of Raw 264.7 cells stimulated by LPS (100 ng/ml, pET-28a empty (the production of E. Coli induced by pET-28a empty vector without CsHscB open reading frame), the elution buffer (containing unbound proteins in CsHscB solution during liquid chromatography) and the purified rCsHscB (20 μg/ml) for 24 h. Quantitative data are representative of mean ± SEM of at least three independent experiments. Compared with indicated group, * P<0.05, **P<0.01, ***P<0.001. (TIF) [file pntd.0008643.s004.tif]
